# Bendamustine Conditioning Skews Murine Host DCs Toward Pre-cDC1s and Reduces GvHD Independently of Batf3

**DOI:** 10.3389/fimmu.2020.01410

**Published:** 2020-07-16

**Authors:** Megan S. Molina, Jessica Stokes, Emely A. Hoffman, Jelena Eremija, Yi Zeng, Richard J. Simpson, Emmanuel Katsanis

**Affiliations:** ^1^Department of Immunobiology, University of Arizona, Tucson, AZ, United States; ^2^Department of Pediatrics, University of Arizona, Tucson, AZ, United States; ^3^Department of Pathology, University of Arizona, Tucson, AZ, United States; ^4^Department of Nutritional Science, University of Arizona, Tucson, AZ, United States; ^5^University of Arizona Cancer Center, University of Arizona, Tucson, AZ, United States; ^6^Department of Medicine, University of Arizona, Tucson, AZ, United States

**Keywords:** graft-vs.-host disease, BMT, conditioning, dendritic cells, bendamustine

## Abstract

Graft-versus-host disease (GvHD) remains the second leading cause of death in allogeneic hematopoietic stem cell transplantation recipients, highlighting the need for improved preventative strategies. Our laboratory has previously demonstrated in an experimental bone marrow transplantation (BMT) model that bendamustine combined with total body irradiation (BEN+TBI) is a safer alternative to cyclophosphamide with TBI (CY+TBI). The biological mechanisms of action of BEN have not been fully elucidated and likely involve multiple cell populations. Host dendritic cells (DCs) can prime naïve donor T-cells immediately following transplantation, making host DCs critical for the initiation phase of GvHD. We hypothesized that BEN+TBI conditioning favorably alters host DC composition to reduce GvHD. We demonstrate that host DCs treated with BEN+TBI induce less allogeneic T-cell proliferation than those conditioned with CY+TBI. We further show that BEN+TBI conditioning results in greater total numbers of all host DC subsets but with a more favorable composition compared to CY+TBI with significantly larger proportions of type 1 conventional DCs (cDC1), a highly regulatory DC subset capable of suppressing GvHD. Our studies using recipient Batf3 KO mice indicate that CD8α+ cDC1s are largely dispensable for the reduced GvHD following BEN+TBI conditioning. We found a higher frequency of host pre-cDC1s with BEN+TBI conditioning in both wild-type (WT) and Batf3 KO mice, which was inversely associated with GvHD. Additionally, we observed that BEN treatment results in greater expression of Flt3 receptor (CD135) on host DCs compared to CY, potentially contributing to the skewing of host DCs toward cDC1s. Further, BEN+TBI conditioning results in host cDCs with greater expression of PIR-B, an inhibitory receptor capable of preventing lethal GvHD. We conclude that BEN+TBI is a safer alternative to CY+TBI, resulting in a greater frequency of host pre-cDC1s and limiting GvHD.

## Introduction

Graft-versus-host disease (GvHD) remains a significant complication of allogeneic hematopoietic cell transplantation (alloHCT). Efforts to limit GvHD have primarily focused on depleting or modulating donor T-cells through the use of prophylactic post-transplant T-cell suppressing agents. However, these approaches may be associated with risks of allograft rejection and reduced graft-versus-leukemia (GvL) activity (1). There remains a critical need to develop new strategies that limit GvHD without compromising engraftment or GvL. Pre-transplant conditioning regimens not only have direct anti-cancer effects, but can also influence long-term GvHD and GvL. Still, modification of these preparative chemotherapy regimens as a means to limit GvHD and enhance GvL has received little attention.

Previous work from our laboratory has resulted in an ongoing phase I clinical trial investigating the use of bendamustine (BEN) following haploidentical transplant (2–5). The most widely used conditioning regimen in alloHCT for acute lymphoblastic leukemia (ALL) is cyclophosphamide (CY) with total body irradiation (TBI) (6). BEN is traditionally used clinically in chemotherapy-based conditioning regimens for autologous (7–9) and allogeneic (10, 11) transplants, but not in combination with TBI. Our laboratory has shown that replacing CY+TBI with BEN+TBI as pre-transplant conditioning reduces GvHD and improves survival in an MHC-mismatched murine bone marrow transplantation (BMT) model (12). We have previously reported that this difference in GvHD is not due to graft rejection, or a difference in engraftment kinetics. Moreover, we have excluded the possibility that the difference in GvHD is due to conditioning regimen-related toxicity by performing syngeneic BMT, wherein neither BEN nor CY groups exhibit clinical or histological evidence of GvHD (12). Further, we have determined that there are no differences in the intestinal epithelial barrier integrity with BEN vs. CY conditioning, with both groups showing comparable early histological evidence of GvHD (12). Our laboratory has also found that BEN+TBI conditioning results in tolerant T-cells while preserving T-cell dependent GvL (13). Thus, we have reasoned that there are unique immunomodulatory effects of BEN conditioning that provide advantages over CY. Like CY, BEN is an alkylating agent, but is unique in that it also contains a purine analog, conferring anti-metabolite functions that are currently unexplored in an HCT setting (14, 15). We have also shown in a haploidentical mouse model that replacing post-transplant CY with BEN as GvHD prophylaxis limits GvHD while maintaining GvL, further suggesting favorable immunomodulation by BEN (2).

We previously reported that myeloid-derived suppressor cells (MDSCs) are partially responsible for the decreased GvHD seen with BEN+TBI conditioning (12). However, other cell types are likely involved. Host cells are often overlooked in the context of alloHCT due to the fact that they are eliminated by the conditioning regimen. However, host dendritic cells (DCs) persist long enough to stimulate naïve donor T-cells immediately following transplantation and are, therefore, critical in the pathogenesis of GvHD, particularly the initiation phase of GvHD (16–22). There are two main lineages of DCs, plasmacytoid (pDCs) and conventional (cDCs). Of the two lineages, only persistent host cDCs are capable of directly presenting host antigens (Ag) to donor T-cells (23). cDCs exist as either type 1 (cDC1) or type 2 (cDC2) subsets, which primarily prime CD8+ T-cells and CD4+ T-cells, respectively. Batf3-dependent cDC1s have been linked to suppression of GvHD through activation-induced clonal deletion of allospecific donor T-cells (24) as well as superior GvL due to the specialized ability of these cells to capture, process and present tumor Ag to CD8+ T-cells (25–27). Studies using the administration of exogenous Flt3 Ligand (Flt3L) prior to transplantation have highlighted the role that host DCs, particularly host cDC1s, play in limiting GvHD (24, 28). Additionally, on the various DC subsets, greater expression of the stimulatory markers CD80 and CD86 allows for stronger engagement of naïve T-cells (29–36). Equally important in naïve T-cell priming is the expression of inhibitory markers, such as paired immunoglobulin-like receptor B (PIR-B) which has been shown to control lethal GvHD (37). Overall, it appears the relative proportions, numbers, and expression profiles of stimulatory and inhibitory markers of each DC subset in the peri-transplant period are central to the pathogenesis of GvHD.

In this study, we sought to characterize the effect of BEN+TBI on host DC populations to further elucidate the mechanisms by which BEN+TBI conditioning limits GvHD compared to CY+TBI. We demonstrate that BEN+TBI results in a skewed host DC subset composition at the time of transplant toward pre-cDC1s and Batf3-dependent cDC1s *in vivo* and results in DCs with reduced ability to stimulate allogeneic T-cell proliferation *ex vivo*. We reveal that while host Batf3-dependent CD8α+ and CD103+ cDC1s contribute to improved GvHD with BEN+TBI compared to CY+TBI, they are largely dispensable. Reduced GvHD observed with BEN+TBI is associated with higher proportions and absolute numbers of host pre-cDC1s, a Batf3-independent immediate precursor to CD8α+ cDC1s. We further found that BEN treatment results in greater expression of the receptor tyrosine kinase Flt3 (CD135), as well as greater expression of the GvHD-suppressing inhibitory receptor Paired Immunoglobulin-like Receptor B (PIR-B). Altogether, we conclude that BEN+TBI compared to CY+TBI conditioning results in greater murine host pre-cDC1s in a Batf3-independent manner and reduces GvHD.

## Materials and Methods

### Mice

All strains of mice used (BALB/c, C57BL/6 and C.129S-*Batf3*^*tm*1*Kmm*^/J) were age-matched 6–10-week-old females purchased from The Jackson Laboratory (Bar Harbor, ME). Mice were housed in specific pathogen-free conditions and cared for according to the guidelines of the University of Arizona's Institutional Animal Care and Use Committee.

### Drug Preparation and Administration

Cyclophosphamide (Sigma-Aldrich, St. Louis, MO) and bendamustine (SelleckChem, Houston, TX) were reconstituted and diluted as described previously (2). Cyclophosphamide was reconstituted in ddH_2_O to a stock concentration of 50 mg/mL then diluted with sterile saline (General Laboratory Products, Yorkville, IL) for i.p. injection. Bendamustine was reconstituted in dimethyl sulfoxide (Sigma-Aldrich) to a stock concentration of 75 mg/mL, and diluted with sterile phosphate-buffered saline (GE Healthcare Life Sciences) containing 0.2% carboxymethylcellulose and 0.25% polysorbate 80 (Sigma-Aldrich) for i.v. injection.

### BMT Models

BALB/c or Batf3 KO (C.129S-*Batf3*^*tm*1*Kmm*^/J) recipients (H-2^d^) received 40 mg/kg BEN i.v. or 200 mg/kg CY i.p. day −2 and 400 cGy TBI day −1 using a Cesium 137 irradiator, as previously described (12). Day 0, mice received 10^7^ C57BL/6 (H-2^b^) bone marrow (BM) or T-cell depleted bone marrow (TCD-BM) cells with 3 × 10^6^ purified total T-cells (tT) i.v. Mice were monitored daily, weighed twice weekly and percentage of starting weight was calculated. Additionally, mice were scored clinically on skin integrity, fur texture, posture and activity, and cumulative GvHD scores were calculated (38). Moribund mice, including mice with a cumulative score of ≥8 after day +8, were euthanized.

### Preparation of Total T-Cells and T-Cell Depleted BM for BMT

Total T-cells were isolated from naïve C57BL/6 spleens by negative selection using the mouse Pan T-cell Isolation Kit II (Miltenyi Biotec, Auburn, CA), with a purity of >97%. Where TCD-BM is indicated, T-cells were depleted from BM cells using the CD3ε MicroBead Kit (Miltenyi Biotec), with <0.3% CD3ε+ cells remaining (data not shown).

### Isolation of Dendritic Cells for Analysis

Spleens were processed to single cell suspension and splenocytes were counted. Splenic DCs were isolated from conditioned BALB/c spleens by negative selection using the Pan Dendritic Cell Isolation Kit (Miltenyi Biotec) or by positive selection using the CD11c+ MicroBead Kit (Miltenyi Biotec), with purity and yield matching that of the manufacturer's specifications. The Pan DC population isolated includes both CD11c+ and CD11c- fractions (data not shown). CD8α+ cDC1s were isolated from conditioned BALB/c spleens by negative selection to deplete T, B, and NK cells, and then by positive selection using direct labeling of CD8α using the CD8+ Dendritic Cell Isolation Kit (Miltenyi Biotec).

### Flow Cytometry

For engraftment flow, blood was collected by tail tipping. Spleens were processed to single cell suspension and dendritic cells were isolated as described above then counted. Red blood cells were lysed with BD Pharm Lyse buffer (BD Biosciences, San Jose, CA) and flow cytometry was performed as previously reported (39). Fluorescence data were collected using an LSRFortessa cell analyzer (BD Biosciences) and analyzed using FlowJo 2 (Tree Star, Ashland, OR). Antibodies used were anti-mouse H2K^b^ PerCP-eFluor 710 (AF6-88.5.5.3), CD8α PE-Cy7 (53-6.7), CD103 PE (2E7), PIR-B APC (10-1-PIR) (Thermo Fisher, Carlsbad, CA); CD11c FITC (N418), CD11c VioBlue (REA754) (Miltenyi Biotec); B220 Brilliant Violet 510 (RA3-6B2), SIRPα APC/Cy7 (P84), CD24 Pacific Blue (M1/69), CD80 APC (16-10A1), CD86 Alexa Fluor 700 (GL-1), CCR7 PE-Cy5 (4B12) (Biolegend, San Diego, CA); and CD135 PE-CF594 (A2F10.1) (BD Biosciences). Host-type cells are defined as H2K^b^-; plasmacytoid DCs are defined as CD11c+B220+; conventional DCs are defined as CD11c+B220-; cDC1s are defined as CD11c+B220-CD8α+; cDC2s are defined as CD11c+B220-SIRPα+; CD103+ cDC1s are defined as CD11c+B220-CD103+CD8α±; pre-cDC1s are defined as CD11c+B220-CD24^high^CD8α-.

### Mixed Leukocyte Reactions

Mixed leukocyte reactions (MLRs) were conducted and analyzed as previously reported (2). In assays using tritiated-thymidine, T-cells were isolated from spleens of naïve C57BL/6 mice and stimulated with isolated DCs or CD3/CD28 beads as a positive control (Thermo-Fisher Scientific, Waltham, MA). Splenic DCs from naïve or BEN or CY conditioned BALB/c mice with and without TBI were isolated and co-incubated with T-cells for 3 days at 37°C in 7.5% CO_2_ in a 96-well plate. 0.5 μCi of tritiated thymidine was added to each well on day 3 of culture. After an additional 18 h of culture, plates were harvested using a Brandel wash pump harvester (Gaithersburg, MD). T-cell proliferation was measured as counts per minute (CPM) using a MicroBeta2 counter (PerkinElmer, Waltham, MA).

### Suppression Assays

Suppression assays were conducted and analyzed as previously reported (2). In assays using tritiated-thymidine, T-cells were isolated from spleens of naïve C57BL/6 mice and stimulated with CD3/CD28 beads (Thermo Fisher Scientific, Waltham, MA). Splenic DCs from naïve or BEN+TBI or CY+TBI conditioned BALB/c mice were isolated and co-incubated with pre-stimulated T-cells for 3 days at 37°C in 7.5% CO_2_ in a 96-well plate. 0.5 μCi of tritiated thymidine was added to each well on day 3 of culture. After an additional 18 h of culture, plates were harvested using a Brandel wash pump harvester. T-cell proliferation was measured as CPM using a MicroBeta2 counter. For supplemental experiments, T-cells were stained with CellTrace Violet (Invitrogen) immediately following isolation from the spleen and pre-stimulated with CD3/CD28 beads then co-cultured with naïve or BEN+TBI or CY+TBI conditioned DCs. After 4 days of co-incubation, flow cytometry was performed and data was analyzed using Modfit Software (Verity Software House, Topsham, ME) to determine the T-cell proliferation index (PI).

### Flt3 Ligand ELISA

Blood was collected via cardiac puncture, spun down at 10,000 g for 10 min and plasma was collected and stored at −20°C. Plasma was used in an ELISA per the manufacturer's protocol to determine plasma levels of Flt3 Ligand (R&D Systems, Minneapolis, MN).

### Statistical Analysis

Kaplan-Meier curves were analyzed by Mantel-Cox log-rank test to determine survival percentages and differences between groups. Mann-Whitney tests were used to determine significant differences in cell counts, percentages, proliferation, weight, and GvHD scores. One-way ANOVA and Tukey's *post-hoc* tests were used to determine fold-change differences among DC populations. *P*-values < 0.05 were considered statistically significant.

## Results

### BEN+TBI Results in Host DCs Less Stimulatory of Alloreactive T-Cells and Improves GvHD Compared to CY+TBI Conditioning

**Figure 1:**
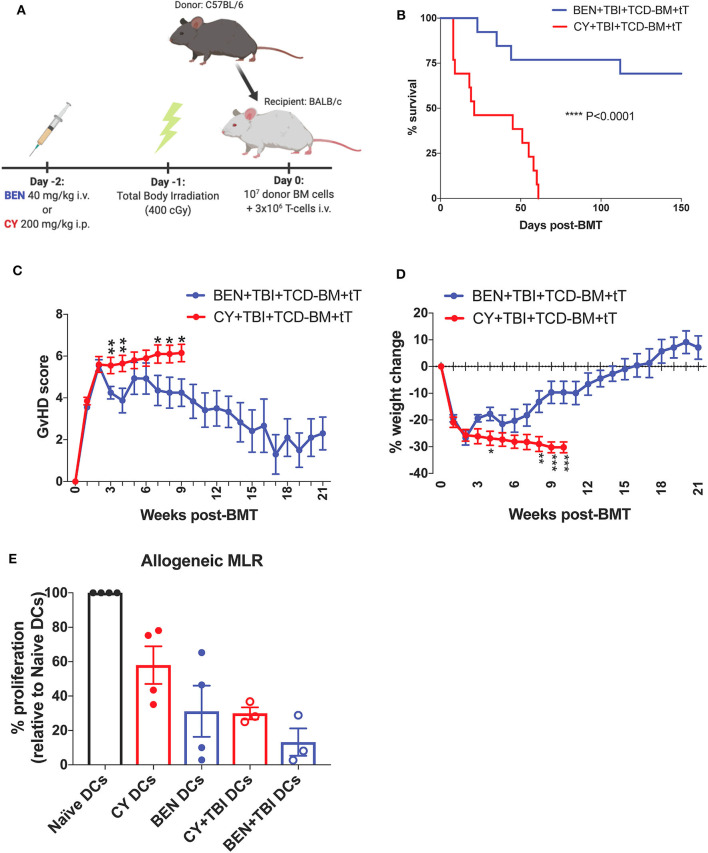
BEN+TBI compared to CY+TBI conditioning results in host DCs less stimulatory of alloreactive T-cells and improves GvHD. **(A)** BALB/c recipient mice received 40 mg/kg BEN i.v. or 200 mg/kg CY i.p. on day −2, 400 cGy TBI on day −1 and 10^7^ T-cell depleted bone marrow (TCD-BM) with 3 × 10^6^ purified T-cells (tT) on day 0. Created with Biorender.com. **(B)** Pooled survival data from 3 experiments are shown, *n* = 15 mice/group. A log-rank Mantel-Cox test was used to determine significance. ****P < 0.0001. **(C)** The weekly average of the mean clinical GvHD score per group is shown with SEM. **(D)** The weekly mean percent weight change from the starting weight with SEM is shown. Pooled data from 3 experiments are shown, *n* = 15 mice/group. Multiple *t*-tests were used to determine significance. *P < 0.05, **P < 0.01, ***P < 0.001. **(E)** BALB/c recipient mice received 40 mg/kg BEN i.v. or 200 mg/kg CY i.p. on day −2 and 400 cGy TBI on day −1. Splenic DCs from naïve or BEN or CY treated mice with or without TBI were isolated by MACS negative selection on day 0 and used as stimulators of allogeneic T-cells. MLRs were plated at a stimulator to responder ratio of 1:10. T-cell proliferation was assessed by tritiated-thymidine uptake after 4 days of co-culture and shown as percent proliferation (relative to Naïve DCs) with SEM. Pooled data from 4 experiments are shown, *n* = 3–4 mice/group. Mann-Whitney unpaired *t*-tests were used to determine significance (BEN vs. CY *P* = 0.34; BEN+TBI vs. CY+TBI *P* = 0.40).

To mimic clinical BMT, recipient mice were conditioned with BEN or CY supplemented with TBI as outlined in Figure 1A. In a severe GvHD model (10^7^ TCD-BM + 3 × 10^6^ total T-cells), BEN+TBI conditioning results in significantly improved survival (Figure 1B) with lower clinical GvHD scores and reduced weight loss (Figures 1C,D) compared to CY+TBI. Complete engraftment was achieved with both conditioning regimens (Supplemental 1). We have previously shown that syngeneic controls exhibited complete engraftment and survival with no signs of GvHD, indicating that deaths are not due to toxicity from the conditioning regimens (2). As host APCs are well-known to play a significant role in GvHD pathogenesis, we hypothesized that BEN+TBI conditioning alters host DCs in a way that attenuates acute GvHD pathogenesis. To test this, we performed an MLR using DCs from BEN- or CY-conditioned mice with or without TBI as stimulators of allogeneic T-cells. We found that BEN-conditioned DCs have reduced capacity to stimulate allogeneic T-cell proliferation compared to naïve or CY-conditioned DCs (Figure 1E), with or without the addition of TBI, although this was not statistically significant. To exclude the possibility that conditioned DCs may have been differentially necroptotic or apoptotic in culture, we determined viability at the beginning of culture by Trypan blue staining and performed a Propidium Iodide and Annexin V staining at the end of co-culture and determined that there were no differences in viability between BEN+TBI and CY+TBI conditioned DCs at either timepoint (data not shown). These results support our hypothesis that BEN+TBI conditioning attenuates the capacity of host DCs to stimulate alloreactive T-cell proliferation, albeit mildly.

### A Higher Ratio of Host Plasmacytoid to Conventional DCs Remain After BEN+TBI Conditioning Compared to CY+TBI Conditioning

**Figure 2:**
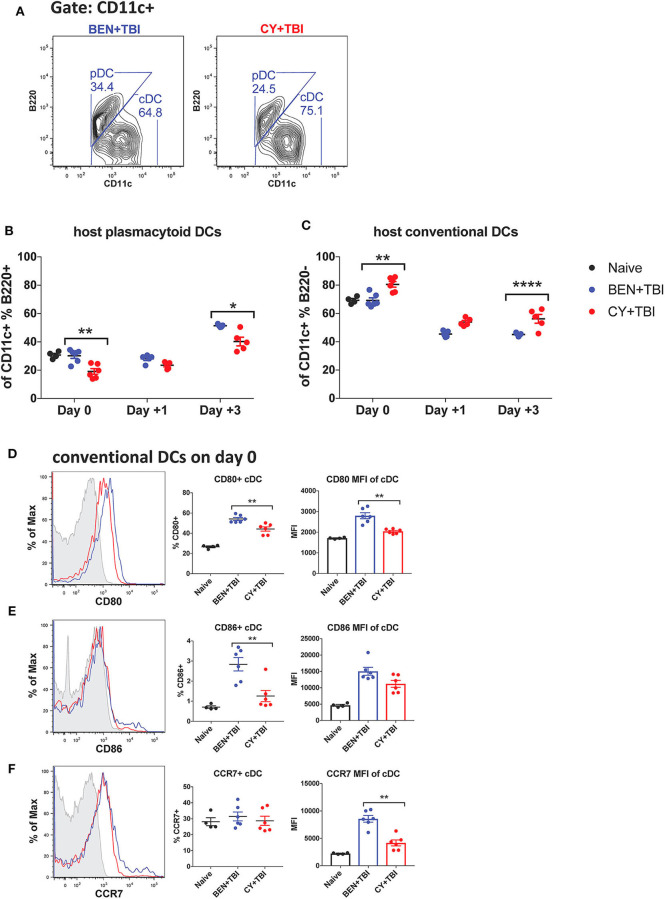
Higher ratios of host plasmacytoid to conventional DCs remain and conventional DCs have a higher expression of CD80 and CD86 after BEN+TBI compared to CY+TBI conditioning. BALB/c mice received 40 mg/kg BEN i.v. or 200 mg/kg CY i.p. day −2 and 400 cGy TBI on day −1. Some groups were transplanted on day 0 with 10^7^ BM + 3 × 10^6^ total T-cells for post-transplant analysis of DCs. On day 0, +1 or +3, spleens were collected and DCs were isolated by MACS negative selection and counted before analysis by flow cytometry. Naïve mice were used as controls. **(A)** Representative flow cytometry gating of conventional (CD11c+B220–) and plasmacytoid (CD11c+B220+) DC subsets are shown. Of total host CD11c+ DCs, **(B)** percent pDCs and **(C)** percent cDCs were quantified on day 0, +1 and +3. Representative histograms, percent and MFI among cDCs positively expressing the activation markers **(D)** CD80, **(E)** CD86 and **(F)** CCR7 are shown, with fluorescence minus one (FMO) control shown in gray, BEN+TBI shown in blue and CY+TBI shown in red. Pooled data from 2 experiments are shown, *n* = 4–6 mice per group per time point. Mann-Whitney unpaired *t*-tests were used to determine significance with SEM shown. *P < 0.05, **P < 0.01, ****P < 0.0001.

**Figure 3:**
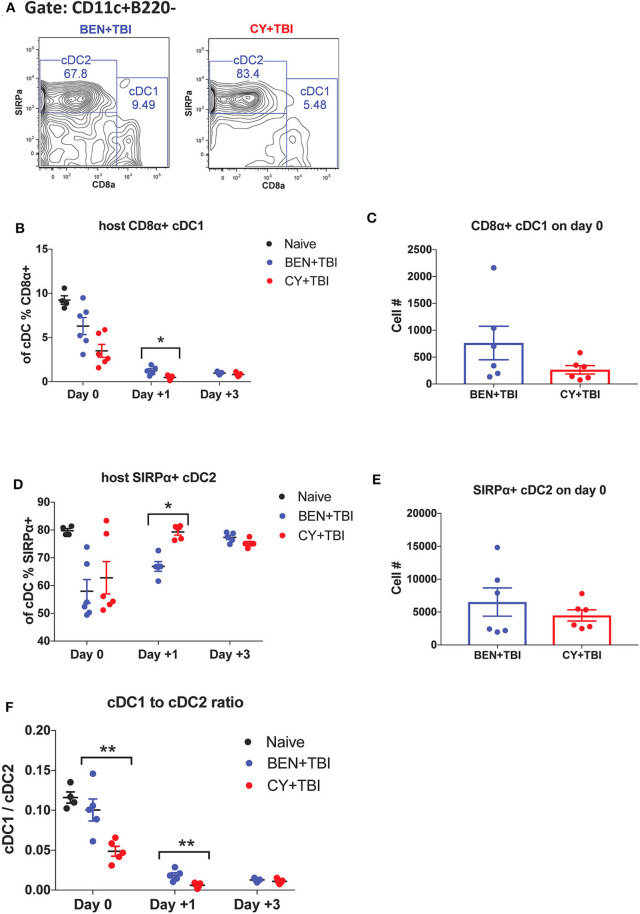
BEN+TBI compared to CY+TBI conditioning results in higher proportions of CD8α+ cDC1s. Data shown is from the same mice as in Figure 2. **(A)** Representative flow cytometry gating of splenic CD8α+ cDC1 and SIRPα+ cDC2 populations on day 0 with BEN+TBI or CY+TBI. Quantification of **(B)** percent CD8α+ cDC1s on days 0, +1 and +3 and **(C)** total number of CD8α+ cDC1s on day 0 are shown for BEN+TBI and CY+TBI groups. Quantification of **(D)** percent SIRPα+ cDC2s on days 0, +1 and +3 and **(E)** total number of SIRPα+ cDC2s on days 0 are shown for BEN+TBI and CY+TBI groups. Naïve mice were used as controls. **(F)** Ratio of favorable CD8α+ cDC1s to unfavorable SIRPα+ cDC2s on day 0, +1 and +3 is shown. Pooled data from 2 experiments are shown, *n* = 4–6 mice per group per timepoint. Mann-Whitney unpaired *t*-tests were used to determine significance with SEM shown. *P < 0.05, **P < 0.01.

**Figure 4:**
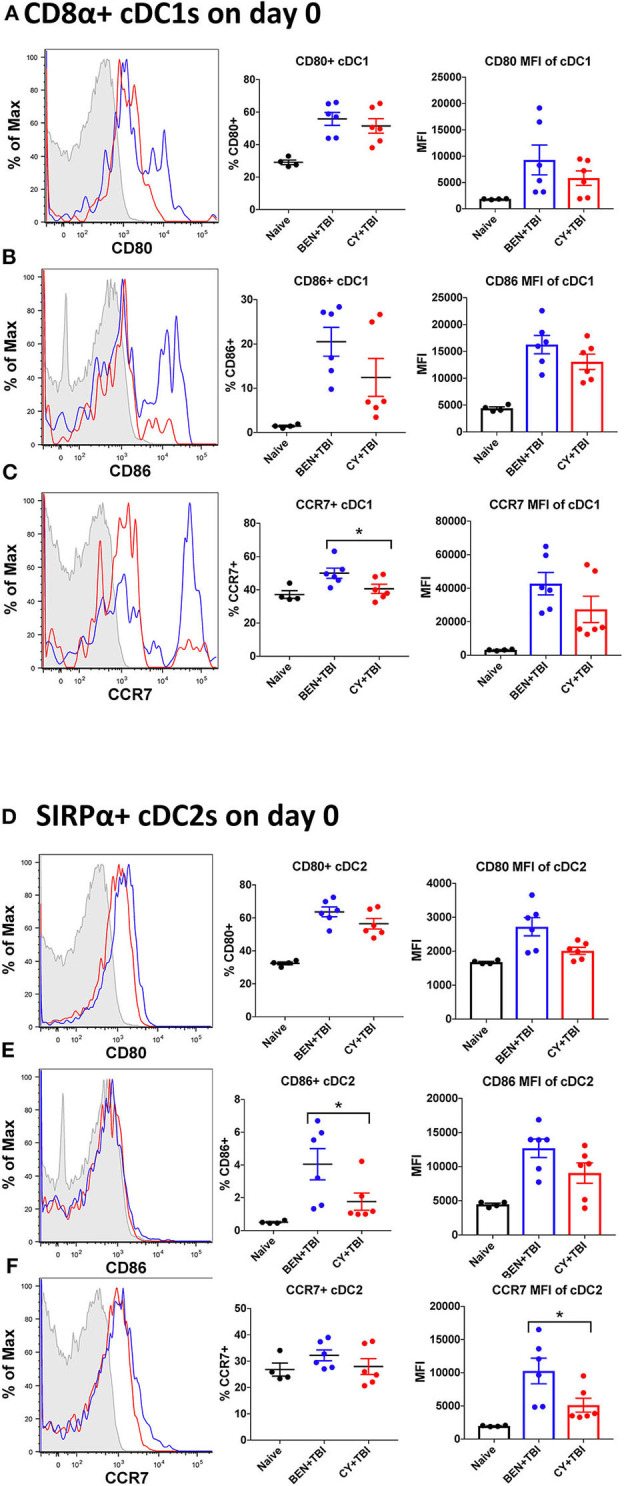
BEN+TBI compared to CY+TBI conditioning results in more highly activated cDCs with greater migratory capacity. Data shown is from the same mice as in Figure 2. Representative histograms, percent and MFI among cells positively expressing the activation markers CD80, CD86 and CCR7 are shown for **(A–C)** splenic CD8α+ cDC1s and **(D–F)** splenic SIRPα+ cDC2s, with FMO control shown in gray, BEN+TBI shown in blue and CY+TBI shown in red. Pooled data from 2 experiments are shown, *n* = 4–6 mice per group per timepoint. Mann-Whitney unpaired *t*-tests were used to determine significance with SEM shown. *P < 0.05.

**Figure 5:**
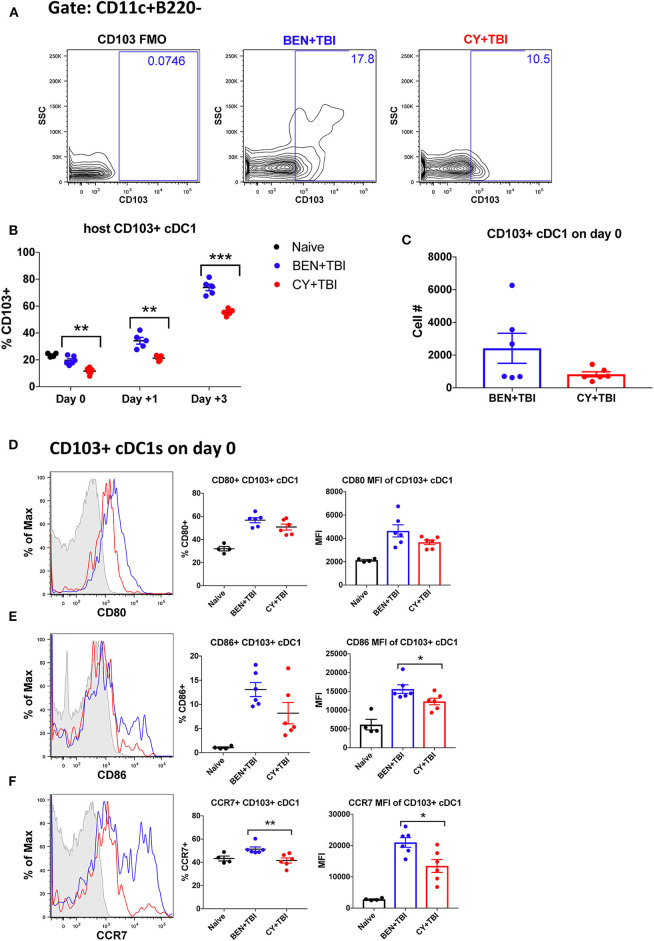
BEN+TBI compared to CY+TBI conditioning results in higher proportions of CD103+ cDC1s and robust accumulation of pre-cDC1s. Data shown is from the same mice as in Figure 2. **(A)** Representative flow cytometry gating of the splenic CD103+ cDC1 population on day 0, showing the FMO control. **(B)** Quantification of percent CD103+ cDC1s on days 0, +1 and +3 are shown from BEN+TBI or CY+TBI conditioned mice. Naïve mice were used as controls. **(C)** Total cell numbers of CD103+ cDC1 on day 0 are shown. Representative histograms, percent and MFI among cells positively expressing the activation markers **(D)** CD80, **(E)** CD86 and **(F)** CCR7 are shown for CD103+ cDC1s, with FMO control shown in gray, BEN+TBI shown in blue and CY+TBI shown in red. Pooled data from 2 experiments, *n* = 4–6 mice per group per time point. Mann-Whitney unpaired *t*-test was used to determine significance with SEM shown. *P < 0.05, **P < 0.01, ***P < 0.001.

**Figure 6:**
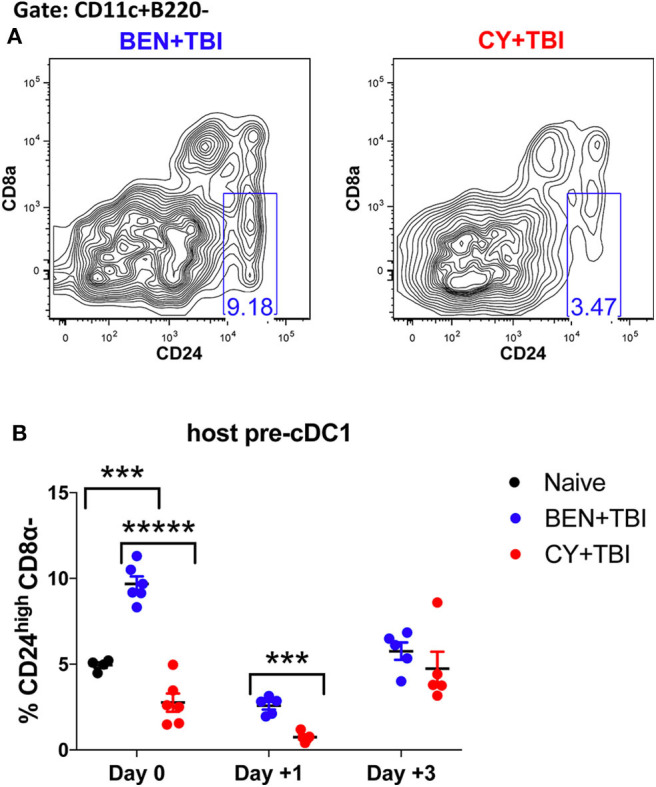
BEN+TBI conditioning results in robust accumulation of pre-cDC1s. Data shown is from the same mice as in Figure 2. **(A)** Representative flow cytometry gating of the splenic CD24^high^CD8α- pre-cDC1 population on day 0 is shown. **(B)** Quantification of percent pre-cDC1s on days 0, +1 and +3 are shown for BEN+TBI and CY+TBI groups. Naïve mice were used as controls. Pooled data from 2 experiments, *n* = 4–6 mice per group per time point. Mann-Whitney unpaired *t*-test was used to determine significance with SEM shown. ***P < 0.001, *****P < 0.00001.

We next sought to characterize the overall composition of host DC subsets and their activation status following BEN+TBI compared to CY+TBI. Conditioning results in significant and extensive epithelial tissue necrosis (data not shown), making isolation of DCs from GvHD target-tissues such as the intestines at these early time points technically difficult. Therefore, to assess the relative abundance of the two major host DC lineages in the peri-transplant period, mice were conditioned with BEN+TBI or CY+TBI and splenic DCs were collected either on day 0 (prior to transplant) or post-BMT on day +1 or +3, as depicted in Supplemental 2. Isolated splenic DCs were counted and analyzed by flow cytometry. DC subset data shown herein (Figures 2–6) are from the same subjects. There were no significant differences in the absolute number or percent yield of DCs isolated from spleen between BEN+TBI and CY+TBI groups on day 0 (Supplemental 3). Flow cytometric analysis confirmed that H2K^b^- host cells comprised >50% of all splenic DCs through day +3 post-transplant (Supplemental 4). The identifying markers used to characterize each DC subset and accompanying gating strategies are shown in Supplementals 5, 6 (29, 40). Among isolated splenic DCs, there was no difference in percent host CD11c+ DCs between BEN+TBI and CY+TBI treated groups on day 0, +1 or +3 (data not shown). The mice receiving BEN+TBI conditioning had a significantly higher percentage of host pDCs (Figures 2A,B) and a significantly lower percentage of host cDCs (Figures 2A,C) compared to CY+TBI on day 0 and day +3.

### Conventional DCs Have a Higher Expression of CD80 and CD86 After BEN+TBI Compared to CY+TBI Conditioning

We were additionally interested in assessing host cDC expression of the co-stimulatory molecules CD80 and CD86 and chemokine receptor CCR7 on day 0. BEN+TBI conditioning yielded a significantly higher percentage of CD80+ and CD86+ cDCs compared to CY+TBI, with a significantly greater CD80 MFI (Figures 2D,E) of CD80+ cDCs. BEN+TBI conditioning additionally resulted in a significantly greater CCR7 MFI on CCR7+ cDCs compared to CY+TBI (Figure 2F). In summary, our results indicate that BEN+TBI conditioning results in proportionally more pDCs compared to cDCs than CY+TBI, and in more highly activated cDCs with greater potential to migrate to secondary lymphoid organs compared to CY+TBI.

### BEN+TBI Compared to CY+TBI Conditioning Results in Higher Proportions of CD8α+ cDC1s

cDCs resistant to conditioning are capable of priming naïve donor T-cells and guiding their effector functions (23, 40). In mice, CD8α+ cDC1s are adept at cross-presentation and priming of CD8+ T-cells while the SIRPα+ cDC2s prime CD4+ T-cells. Several reports have found that CD8α+ cDC1s play an important role in suppressing alloreactive T-cell responses and limiting GvHD through activation-induced clonal deletion of allospecific donor T-cells (25–27). Therefore, we evaluated the two host cDC subsets in BEN+TBI vs. CY+TBI conditioning. Representative flow plots of cDC1 and cDC2 populations on day 0 are depicted in Figure 3A, demonstrating differences between BEN+TBI and CY+TBI. The CD8α-SIRPα- DCs are considered pre-cDCs that have not yet committed to either of the two cDC lineages. Quantification of these flow cytometry plots shows significantly more CD8α+ cDC1s with BEN+TBI conditioning than with CY+TBI (Figure 3B), and significantly fewer SIRPα+ cDC2s (Figure 3D) on day +1. The absolute numbers of CD8α+ cDC1s (Figure 3C) and SIRPα+ cDC2s (Figure 3E) were not different on day 0. Given that total cDCs have been reported to exacerbate GvHD, whereas CD8α+ cDC1s are highly effective suppressors of GvHD, we evaluated the ratio of CD8α+cDC1 to SIRPα+cDC2 in each mouse conditioned with BEN+TBI or CY+TBI. We found that the ratio of favorable cDC1s to unfavorable cDC2s was significantly higher in BEN+TBI mice on days 0 and +1 (Figure 3F).

### BEN+TBI Compared to CY+TBI Conditioning Results in More Highly Activated cDCs With Greater Migratory Capacity

Both BEN+TBI conditioned cDC subsets trended toward higher percent positive and MFI of the co-stimulatory molecules CD80 and CD86 on day 0 (Figures 4A–F), with cDC2s demonstrating a significantly higher percent of CD86+ cells (Figure 4E). Additionally, both BEN+TBI conditioned cDC subsets trended toward a higher percent positive and MFI of CCR7, reaching significance in cDC1 percent positive (Figure 4C) and in cDC2 MFI (Figure 4F). Overall, we observed more activated, migratory cDCs and a significantly greater proportion of CD8α+ cDC1s in BEN+TBI conditioned mice compared to CY+TBI.

### BEN+TBI Compared to CY+TBI Conditioning Results in Higher Proportions of CD103+ cDC1s

We next sought to evaluate the mobile, non-lymphoid-residing counterpart of CD8α+ cDC1s, CD103+ cDC1s. CD103+ cDC1s are functionally equivalent to CD8α+ cDC1s, with both subsets requiring the Batf3 transcription factor for development, expressing the same set of pattern-recognition receptors and displaying the same Ag-processing and presentation capabilities (40). The major difference is that CD8α+ cDC1s reside within secondary lymphoid organs while CD103+ cDC1s reside primarily in skin and intestines and migrate to secondary lymphoid organs upon activation (31, 40). Therefore, we evaluated CD103+ cDC1s in the spleen, bearing in mind that CD103+ cDC1s may co-express CD8α+ and may overlap in our percentage analyses. We observed a significantly greater percentage of CD103+ cDC1s in BEN+TBI conditioned mice compared to CY+TBI on day 0, +1 and +3 (Figures 5A,B), with a trend toward higher absolute number of CD103+ cDC1s in BEN+TBI compared to CY+TBI conditioning (Figure 5C). BEN+TBI conditioning resulted in a trend toward increased expression of CD80 and CD86 that reached significance in the CD86 MFI of CD86+ CD103+ cDC1s on day 0 (Figures 5D,E). Further, when we evaluated the migratory capacity of these CD103+ cDC1s we found that BEN+TBI conditioning resulted in a significantly greater percent expression and MFI of CCR7 compared to CY+TBI (Figure 5F). Altogether, these results suggest that BEN+TBI conditioning favors the persistence, activation and migration of host Batf3-dependent CD8α+ and CD103+ cDC1s compared to CY+TBI.

### BEN+TBI Conditioning Results in Robust Accumulation of Pre-cDC1s

It remains unclear whether BEN+TBI conditioning spares host cDC1s, alters their recruitment to or from the spleen, or alters the host microenvironment to increase the differentiation of cDC1s *in situ*. To try to evaluate the possibility that BEN+TBI is promoting cDC1 development, we quantified the immediate precursor to CD8α+ cDC1s, termed “pre-cDC1s”, in the spleen. The pre-cDC1s become committed to the CD8α+ cDC1 lineage in the bone marrow and then mobilize to the spleen and other peripheral tissues to differentiate into mature DCs (29, 41). This precursor population is identified by a lack of CD8α and the high expression of CD24, which is a membrane glycoprotein that senses damage-associated molecular patterns (DAMPs) (42). Representative flow plots show distinct differences in pre-cDC1 populations between BEN+TBI and CY+TBI on day 0 (Figure 6A). There are significantly higher proportions of pre-cDC1s with BEN+TBI compared to CY+TBI conditioning, as well as naïve mice, on day 0 (Figure 6B). This significant difference was maintained through day +1 post-transplant. In contrast, we did not observe a consistent difference or trend in pre-cDC2s following BEN+TBI and CY+TBI conditioning (data not shown). These findings indicate that BEN+TBI conditioning may promote the commitment of DC progenitors to the cDC1 lineage.

### BEN+TBI Compared to CY+TBI Conditioned cDC1s Have a More Suppressive Function *ex vivo*

**Figure 7:**
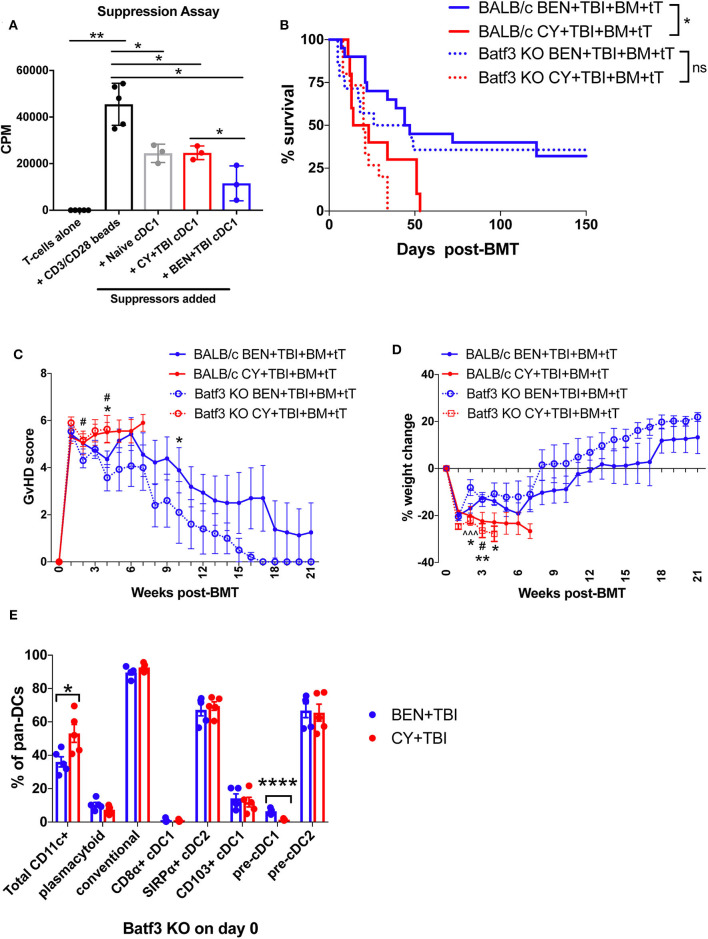
Batf3-dependent CD8α+ cDC1s are more suppressive following BEN+TBI compared to CY+TBI conditioning, but are not the only cell type contributing to reduced GvHD. BALB/c mice received 40 mg/kg BEN or 200 mg/kg CY on day −2 and 400 cGy on day −1. **(A)** On day 0, splenic CD8α+ cDC1s were isolated by magnetic bead isolation and plated in a suppression assay in a 96-well plate with T-cells pre-stimulated with CD3/CD28 beads. 0.5 μCi of tritiated-thymidine was added to each well of cultures on day 3 and T-cell proliferation was quantified as counts per minute (CPM) on day 4. CD8α+ cDC1s from naïve mice were used as a control. Data is from 1 experiment, *n* = 3 mice per group. Mann-Whitney unpaired *t*-tests were used to determine significance with SEM shown. *P < 0.05; **P < 0.01. **(B–D)** BALB/c or Batf3 KO recipient mice received 40 mg/kg BEN i.v. or 200 mg/kg CY i.p. on day −2, 400 cGy TBI on day −1 and 10^7^ BM with 3 × 10^6^ total T-cells (tT) on day 0. **(B)** Pooled survival data from 3 experiments are shown, *n* = 15–20 mice/group. A log-rank Mantel-Cox test was used to determine significance. *P < 0.05. **(C)** The weekly average of the mean clinical GvHD score per group is shown with SEM. **(D)** The weekly mean percent weight change from the starting weight is shown with SEM. Multiple *t*-tests were used to determine significance. *indicates significance between BALB/c BEN+TBI and BALB/c CY+TBI. ^#^Indicates significance between Batf3 KO BEN+TBI and Batf3 KO CY+TBI. ^∧^indicates significance between BALB/c BEN+TBI and Batf3 KO BEN+TBI. **(E)** Batf3 KO mice received 40 mg/kg BEN i.v. or 200 mg/kg CY i.p. on day −2, 400 cGy TBI on day −1 and splenic pan-DCs were isolated by magnetic separation on day 0 for flow cytometry analysis. The percentage of total CD11c+ DCs among pan-DCs and each DC subset within their respective parent gate, as indicated by the gating strategy in Supplemental Material, in BEN+TBI and CY+TBI conditioned Batf3 KO mice on day 0 is shown. Data is from 1 experiment, *n* = 5 mice per group. Mann-Whitney unpaired *t*-tests were used to determine significance with SEM shown. *P < 0.05, ****P < 0.0001.

We next sought to investigate the function of the CD8α+ cDC1 subset more closely in our model. We performed suppression assays to examine whether BEN+TBI conditioned CD8α+ cDC1s exhibited a regulatory function *ex vivo*. Naïve or BEN+TBI or CY+TBI conditioned CD8α+ cDC1s were used as suppressors of T-cells stimulated with CD3/CD28 beads. Isolation purity of CD8α+ cDC1s is shown (Supplemental 7A). When BEN+TBI conditioned cDC1s were added to the stimulated T-cell cultures, there was significantly less T-cell proliferation, measured by tritiated-thymidine uptake, compared to either naïve or CY+TBI conditioned cDC1s (Figure 7A). A similar suppression assay performed by flow cytometric analysis of CellTrace Violet dilution corroborates this finding (Supplemental 7B). These data indicate that BEN+TBI conditioning results in CD8α+ cDC1s with regulatory abilities, capable of suppressing T-cell proliferation to a greater extent than CD8α+ cDC1s conditioned with CY+TBI.

### Host Batf3-Dependent cDC1s Contribute to but Are Not Required for the Reduction of GvHD Following BEN+TBI Conditioning

To better understand the requirement of host cDC1s as a mechanism by which BEN+TBI reduces GvHD, we utilized Batf3 KO mice as BMT recipients alongside wild-type (WT) BALB/c mice. Consistent with reports, Batf3 KO mice are devoid of CD8α+ cDC1s (Supplemental 8A) and significantly deficient in the non-lymphoid residing CD103+ cDC1s (Supplemental 8B) (43, 44). We hypothesized that BEN+TBI promotes regulatory host cDC1s to achieve reduced GvHD and, therefore, expected BEN+TBI conditioned Batf3 KO recipients to exhibit significantly worse GvHD than BEN+TBI conditioned WT BALB/c recipients. Surprisingly, we did not observe significant differences in survival, clinical GvHD score or weight loss between WT and Batf3 KO groups in either BEN+TBI or CY+TBI conditioned groups (Figures 7B–D), with complete donor engraftment confirmed in this model (Supplemental 8C). This indicates that BEN+TBI conditioning remains a safe regimen with limited GvHD mortality and morbidity, even in the absence of cDC1s. However, the difference in survival between BEN+TBI and CY+TBI was trending but no longer significant when utilizing the Batf3 KO recipients (*P* = 0.1449) (Figure 7B). Although the Batf3-dependent DC subsets appear to contribute to the overall advantage of BEN+TBI over CY+TBI, these results indicate that host-type CD8α+ cDC1s are unessential for improved GvHD with BEN+TBI over CY+TBI, indicating that other host cell subsets also play a role.

Given these unexpected results, we sought to characterize the effect of BEN+TBI conditioning on host DC composition in Batf3 KO mice. Batf3 KO mice had overall fewer absolute numbers of DCs compared to BALB/c WT mice. However, we did not find statistical differences between BEN+TBI and CY+TBI conditioning in the total number or percent yield of isolated splenic DCs on day 0 (data not shown). Flow cytometry revealed that BEN+TBI conditioning results in significantly lower percent total CD11c+ cells compared to CY+TBI in the isolated pan-DCs (Figure 7E). As expected, we found negligible percentages of CD8α+ cDC1s. Among the DC subsets in conditioned Batf3 KO mice, only the pre-cDC1 population was significantly higher in BEN+TBI compared to CY+TBI (Figure 7E). This finding is consistent with our results in BEN+TBI conditioned BALB/c mice in Figure 6B in which the pre-cDC1 population was the most significantly elevated on day 0 compared to CY+TBI.

### BEN+TBI Results in Greater Numbers of Pre-cDC1s in Both BALB/c and Batf3 KO Mice Compared to CY+TBI Conditioning

**Figure 8:**
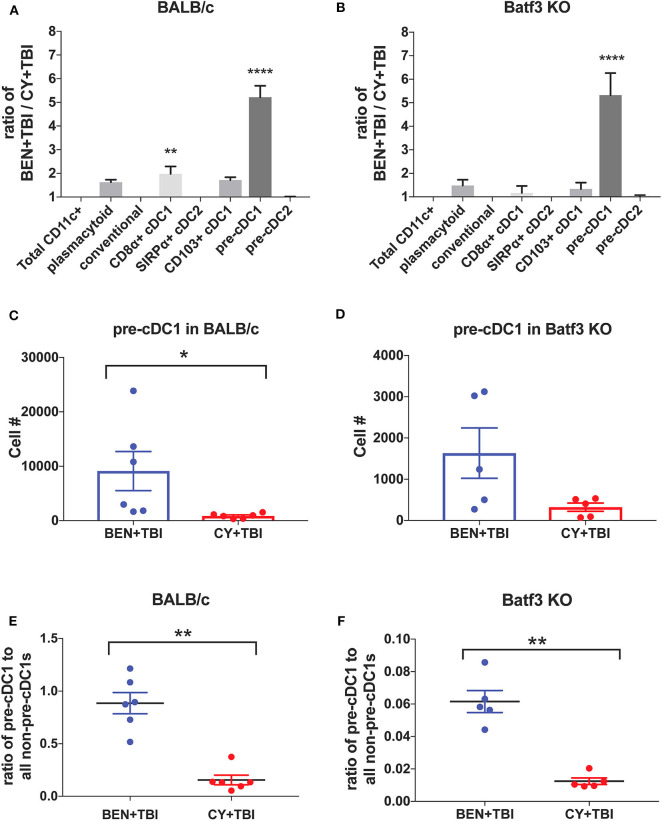
BEN+TBI compared to CY+TBI conditioning results in greater numbers of pre-cDC1s in both BALB/c and Batf3 KO mice. **(A)** The ratio change of each DC subset within BEN+TBI conditioned BALB/c mice compared to CY+TBI conditioned BALB/c mice on day 0 is shown. Data is from 2 experiments, *n* = 6 mice per group. One-way ANOVA and Tukey's *post-hoc* test was used to determine significance compared to total CD11c+ DCs with SEM shown. ****P < 0.0001. **(B)** The ratio change of each DC subset within BEN+TBI conditioned Batf3 KO mice compared to CY+TBI conditioned Batf3 KO mice on day 0 is shown. Data is from 1 experiment, *n* = 5 mice per group. One-way ANOVA and Tukey's *post-hoc* test was used to determine significance compared to total CD11c+ DCs with SEM shown. ****P < 0.0001. **(C)** The total number of splenic pre-cDC1s in BEN+TBI and CY+TBI conditioned BALB/c mice on day 0 is shown. Data is pooled from 2 experiments, *n* = 6 mice per group. Mann-Whitney unpaired *t*-tests were used to determine significance with SEM shown. *P < 0.05. **(D)** The total number of splenic pre-cDC1s in BEN+TBI and CY+TBI conditioned Batf3 KO mice on day 0 is shown. Data is pooled from 2 experiments, *n* = 6 mice per group. Mann-Whitney unpaired *t*-tests were used to determine significance with SEM shown (*P* = 0.098). **(E)** The ratio of pre-cDC1 to all non-pre-cDC1s in BEN+TBI and CY+TBI conditioned BALB/c mice on day 0 is shown. Data is pooled from 2 experiments, *n* = 6 mice per group. Mann-Whitney unpaired *t*-tests were used to determine significance with SEM shown. **P < 0.01. **(F)** The ratio of pre-cDC1 to all non-pre-cDC1s in BEN+TBI or CY+TBI conditioned Batf3 KO mice on day 0 is shown. Data is pooled from 2 experiments, *n* = 6 mice per group. Mann-Whitney unpaired *t*-tests were used to determine significance with SEM shown. **P < 0.01.

To further evaluate the similarities between the effects of BEN+TBI conditioning on BALB/c and Batf3 KO mice, we calculated the ratios of each DC subset with BEN+TBI conditioning compared to CY+TBI. In both BALB/c (Figure 8A) and Batf3 KO mice (Figure 8B) there was a striking ~5-fold higher ratio of pre-cDC1s, the immediate precursor to CD8α+ cDC1s compared to CY+TBI. Pre-cDC1s do not require the Batf3 transcription factor, and are therefore present in our Batf3 KO recipient mice (45). Their role in GvHD and alloreactivity has not been explicitly investigated, though they are reported to function similarly to the CD8α+ cDC1s that have been identified as suppressors of GvHD (24–26, 28). To determine the prevalence and biological relevance of this DC subset in BEN+TBI conditioned mice compared to CY+TBI, we calculated the total cell number as well as the ratio of pre-cDC1s to all other DCs in the spleen on day 0. We observe that BEN+TBI conditioning results in a significantly greater number of pre-cDC1s in the spleens of BALB/c (Figure 8C) and a trend toward more in Batf3 KO mice (Figure 8D). We also found that in both BALB/c (Figure 8E) and Batf3 KO mice (Figure 8F), BEN+TBI conditioning results in a significantly higher ratio of pre-cDC1s to all other DCs compared to CY+TBI. Ultimately, these results indicate a strong association between reduced GvHD and higher numbers of pre-cDC1s, which may play a previously unreported role in suppressing GvHD.

### BEN Compared to CY Treatment Results in Greater Expression of Flt3 Receptor and the Inhibitory Receptor PIR-B on cDCs

**Figure 9:**
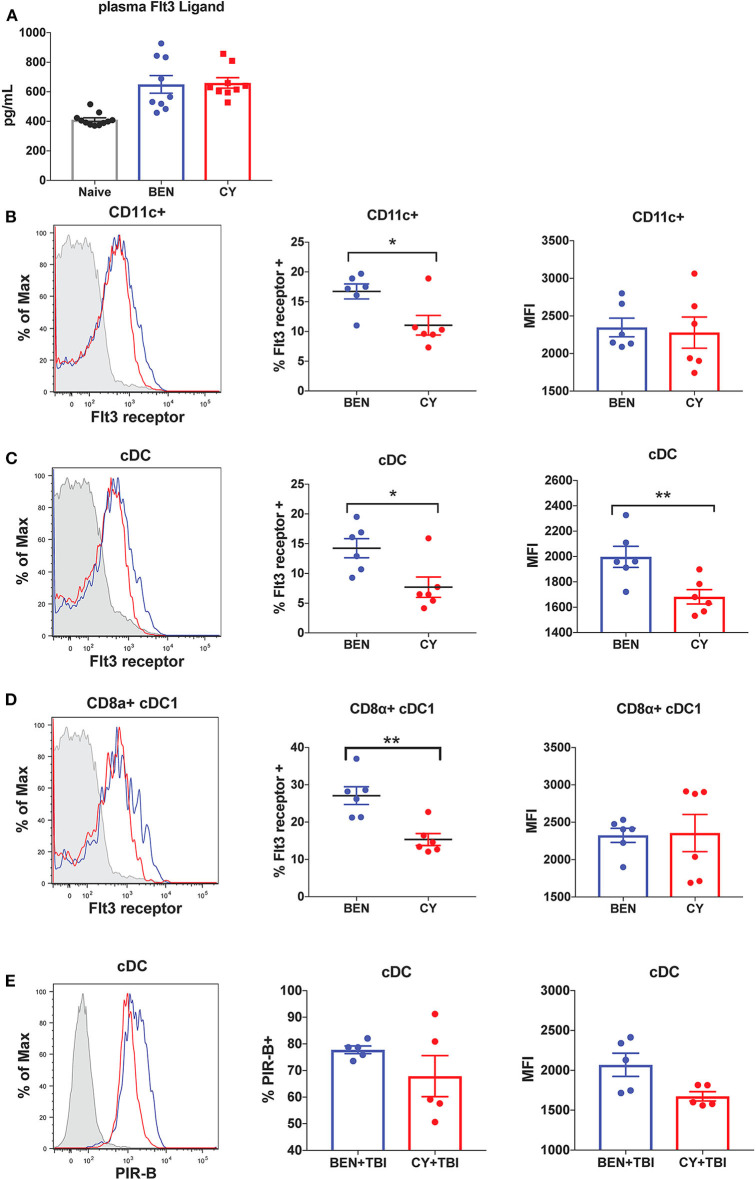
BEN compared to CY treatment results in greater expression of Flt3 receptor and the inhibitory receptor PIR-B on cDCs. BALB/c mice received 40 mg/kg BEN or 200 mg/kg CY on day −2. **(A)** On day 0, blood plasma was collected and used in an ELISA to determine circulating levels of Flt3 Ligand. Naïve mice were used as a control. Mann-Whitney unpaired *t*-tests were used to determine significance with SEM shown. **(B,C)** On day 0, splenic DCs were isolated by MACS negative selection and analyzed by flow cytometry. Representative histograms, percent and MFI of Flt3 receptor on **(B)** CD11c+ DCs **(C)** cDCs (CD11c+B220-) and **(D)** CD8α+ cDC1s positively expressing Flt3 receptor are shown for BEN (blue) and CY (red) treated mice, with FMO control shown in gray. Data is pooled from 2 experiments, *n* = 6 mice per group. Mann-Whitney unpaired *t*-tests were used to determine significance with SEM shown. *P < 0.05, **P < 0.01. **(E)** BALB/c mice received 40 mg/kg BEN or 200 mg/kg CY on day −2 and 400 cGy TBI on day −1. On day 0, splenic DCs were isolated by MACS negative selection and analyzed by flow cytometry. Representative histograms, percent positive (*P* = 0.50) and MFI (*P* = 0.092) of PIR-B on cDCs (CD11c+B220-) are shown for BEN+TBI and CY+TBI conditioned mice. Data is pooled from 2 experiments, *n* = 5 mice per group. Mann-Whitney unpaired *t*-test was used to determine significance with SEM shown.

We next sought to determine the mechanism by which BEN+TBI conditioning promotes the prevalence of cDC1s independently of the Batf3 transcription factor. The DC subset composition observed with BEN+TBI conditioning, associated with increased cDC1s compared to other DC subsets, is consistent with enhanced Flt3 signaling following administration of exogenous Flt3 Ligand (Flt3L) (24, 28). We therefore sought to measure circulating levels of Flt3L to test the hypothesis that BEN achieves greater proportions of cDC1s by enhancing Flt3L signaling. However, when we measured plasma levels of Flt3L on day 0 we found no difference between BEN and CY treated mice that would explain the phenotype (Figure 9A). We additionally evaluated plasma levels of Flt3L on day −1 and again found no difference (data not shown). We, therefore, measured expression levels of the receptor Flt3 (CD135) by flow cytometry and observed that BEN treatment results in significantly greater percent expression of Flt3 among CD11c+ DCs compared to CY conditioning (Figure 9B). Further, we show that conventional DCs have significantly greater expression of Flt3 by percent and MFI with BEN treatment compared to CY treatment (Figure 9C), and this is largely driven by higher expression among CD8α+ cDC1s (Figure 9D).

Administration of exogenous Flt3 Ligand prior to transplant has been shown to significantly reduce GvHD *in vivo*, an effect attributed to robust expansion of CD8α+ cDC1s (24, 28). Murine DCs generated *in vitro* with Flt3L have been shown to closely resemble steady-state DCs and exhibit regulatory function with reduced production of inflammatory cytokines (46). This suggests that Flt3L-driven DCs may acquire a unique transcriptional program compared to DCs receiving other survival and differentiation signals. We therefore sought to determine whether BEN+TBI conditioned DCs exhibited evidence of regulatory function as a result of enhanced Flt3 receptor expression. Paired Immunoglobulin Like Receptor B (PIR-B) delivers inhibitory signals by binding to CL-I on T-cells and is highly expressed on regulatory DCs (37). DCs transfected with PIR-B have been shown to prevent lethal GvHD, and deficiency of PIR-B significantly exacerbates GvHD (37). We therefore measured the expression of PIR-B on conditioned DCs by flow cytometry on day 0. While we found no consistent difference or pattern among CD8α+ cDC1s or pre-cDC1s (data not shown), we found that conventional DCs from BEN+TBI conditioned mice have greater expression of PIR-B by percent and MFI, though this was not significant (Figure 9E). Overall, these data indicate that BEN+TBI conditioning results in host DCs that are more receptive to Flt3L and exhibit greater expression of GvHD-suppressing receptors.

## Discussion

We have extensively studied the C57BL/6 → BALB/c MHC-mismatched BMT model evaluating pre-transplant conditioning regimens. We have previously determined that comparable doses of BEN and CY, based on their respective maximum tolerated dose, induce similar levels of epithelial barrier damage and other host organ toxicities (12). We have also excluded graft rejection and conditioning regimen-induced toxicity as potentially confounding factors by monitoring blood engraftment and using syngeneic BMT controls (12). Both BEN and CY have short half-lives of <4 h, and are eliminated from circulation prior to the time of transplantation (47–49). Therefore, donor cells are not directly exposed to these chemotherapeutic agents, and the improvement in GvHD with BEN+TBI is attributable in part to its effects on host antigen-presenting cells (APCs), particularly host DCs (12). Pre-transplant conditioning regimens result in gradual elimination of host DCs, allowing them to play a critical role in initiation of GvHD immediately following transplantation (19–23). Donor DCs require time to develop from bone marrow progenitors into differentiated DCs, and therefore do not participate in the early induction phase of GvHD (19). It should be noted that our use of H2K^b^- to define host cells on days +1 and +3 post-transplant would not exclude donor DCs that may have lost expression of MHC. In our pre-clinical model, majority of DCs in the spleen on day +3 post-transplant are host-type, offering a window in which they can prime naïve donor T-cells. Further, donor T-cells can generate long-term synapses with host DCs in the spleen and lymph nodes (50), indicating that the composition of splenic host DCs during the peri-transplant period is critical to donor T-cell activation and long-term GvHD outcomes.

We have found that BEN+TBI has a distinct effect on host DCs compared to CY+TBI. We demonstrated that BEN+TBI conditioned DCs stimulate less allogeneic T-cell proliferation *in vitro* compared to CY+TBI conditioned DCs. The differences in splenic host DC composition observed between BEN+TBI and CY+TBI conditioning are summarized in Table 1, along with the reported effect of each DC subset on GvHD upon transfer into recipients. While we observed greater absolute numbers of every DC subset with BEN+TBI conditioning compared to CY+TBI, we found significant changes in the overall composition by percentage. Briefly, we observed higher proportions of pDCs after BEN+TBI conditioning. pDCs are reported to induce GvHD, yet induce tolerance when they are CCR9+ (51, 52). We did not, however, observe any difference in the number of CCR9+ pDCs between BEN+TBI and CY+TBI groups (data not shown). We also observed lower percentage but greater numbers of total cDCs after BEN+TBI conditioning, which are reported to induce GvHD (52). However, when we distinguished between cDC1 and cDC2, we found greater numbers and percentages of both CD8α+ and CD103+ cDC1s in mice receiving BEN+TBI compared to CY+TBI conditioning. While host CD103+ cDC1s have not been explicitly evaluated in the context of GvHD, their lymphoid-residing counterpart, CD8α+ cDC1s, have been widely acknowledged as suppressors of GvHD (24–28). We also observed lower cDC2s in BEN+TBI mice, which can induce GvHD (24, 26, 28, 52). We further documented larger numbers of pre-cDC1s, the immediate precursor to CD8α+ cDC1s, following BEN+TBI conditioning, which have not previously been explicitly evaluated in the context of BMT and GvHD. We also demonstrate numerous incidences of greater expression of the activation markers CD80 and CD86 in BEN+TBI conditioned DCs compared to CY+TBI, indicating that BEN+TBI conditioning results in host cDCs capable of stronger engagement and presentation of host antigen to donor T-cells. We additionally found several incidences of increased expression of CCR7, suggesting CD8α+ and CD103+ cDC1s with greater potential to migrate to lymphoid tissues. However, it is not technically feasible to retrieve sufficient numbers of DCs from lymph nodes or target tissues as there are too few cells following conditioning, limiting our ability to provide direct evidence of enhanced migration. It should also be noted that DCs were isolated without chemical dissociation of spleens, potentially impacting our DC yield and biasing our results.

**Table 1:** Murine DC subset effect on GvHD and summary of results presented as mean cell number and mean percentage.

**Host DC subset**	**Reported effect on GvHD**	**BEN+TBI # (%)**	**CY+TBI # (%)**
pDC	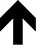 GvHD (39)	5,403 (30.23%)	1,830 (19.12%)
cDC	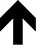 GvHD (39)	12,180 (69.2%)	7,003 (80.52%)
CD8α+ cDC1	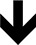 GvHD (17, 40, 41)	763.3 (6.29%)	265.1 (3.49%)
cDC2	Unknown	6,528 (57.97%)	4,490 (62.82%)
CD103+ cDC1	Unknown	2,420 (19.6%)	830.5 (11.66%)
Pre-cDC1	Unknown	9,132 (9.94%)	867 (2.76%)

*↑ Indicates exacerbation of GvHD; ↓ Indicates amelioration of GvHD*.

Murine CD8α+ cDC1s have been widely acknowledged as suppressors of GvHD via activation-induced clonal deletion and exhaustion of allospecific donor T-cells (24–28), warranting further investigation of this subset in the context of BEN+TBI conditioning. We demonstrated that CD8α+ cDC1s conditioned with BEN+TBI exhibit greater suppressive function than those conditioned with CY+TBI. Several groups have shown that Batf3 KO recipients exhibit more severe GvHD (24, 26, 53), while others found that GvHD is unaffected (24, 26, 54). Using our model, survival and clinical GvHD score were comparable in Batf3 KO and WT BALB/c mice receiving BEN+TBI conditioning, indicating that Batf3-dependent CD8α+ cDC1s are expendable to the mitigation of GvHD seen with BEN+TBI. Batf3-dependent CD8α+ cDC1s do appear to play a role in reducing GvHD, however, as there was no longer a significant difference in survival between BEN+TBI and CY+TBI in Batf3 KO mice that was otherwise observed in WT BALB/c mice. Overall, while Batf3-dependent cDC1s do contribute to BEN+TBI conditioning's reduction of GvHD, there appear to be other cellular mechanisms at play.

We also found significantly greater proportions and absolute numbers of pre-cDC1s, the immediate precursor to CD8α+ cDC1s, with BEN+TBI compared to CY+TBI in both WT BALB/c and Batf3 KO mice. This ~5-fold increase in pre-cDC1s with BEN+TBI conditioning was the most striking difference found in any of the host DC subsets and was observed in both strains of mice. Pre-cDC1s are committed to the cDC1 lineage but require the Batf3 transcription factor for their terminal differentiation into mature CD8α+ cDC1s (45). To our knowledge, the role of pre-cDC1s has not been reported in the context of GvHD. However, they express the same pattern recognition receptors as CD8α+ cDC1s, yet have an enhanced lifespan compared to CD8α+ cDC1s (55). Pre-cDC1s have been shown to induce stronger priming of viral-specific CD8+ T-cells compared to mature CD8α+ cDC1s (55). This effect may be attributed to their high expression of CD24, a sensor for damage-associate molecular patterns (DAMPs) that dictates CD8+ T-cell differentiation into effector or memory fates (42). These facets of pre-cDC1 function may prove beneficial in the context of GvHD whereby early priming of CD8+ T-cells against DAMPs may promote tolerance to host antigens. We demonstrate that the prevalence of pre-cDC1s is strongly associated with significant improvements in GvHD and survival achieved with BEN+TBI conditioning, and therefore postulate that pre-cDC1s may have a previously unreported role in limiting GvHD.

There may be an additional advantage of BEN+TBI conditioning's resultant prevalence of pre-cDC1 and CD8α+ cDC1 in the context of viral reactivation. Reactivation of latent viruses, particularly human cytomegalovirus (HCMV), following HSCT is a major cause of morbidity and non-relapse mortality among transplant recipients (56, 57). Numerous studies have determined that Batf3-dependent cDC1s play a critical role in mounting CD8+ T-cell responses against a variety of viruses, including cytomegalovirus (CMV) (58), West Nile virus (WNV) (43), influenza (59, 60), cow pox virus (61), and herpes simplex virus (HSV) (62, 63). Further studies depleting XCR1+ DCs, which include both pre-cDC1s and CD8α+ cDC1s, determined a critical role for these DCs in priming naïve CD8+ T-cell responses and in reactivating memory CD8+ T-cells (64, 65). Therefore, the prevalence of cDC1s in the peri-transplant period with BEN+TBI conditioning could provide the added benefit of protecting against viral reactivation and opportunistic infections. In support of this hypothesis, interim results from an ongoing phase I clinical trial (NCT02996773) to determine the safety of replacing post-transplant CY with post-transplant BEN in haploidentical BMT have shown significantly lower incidence of CMV reactivation and lower CMV viral load with BEN compared to CY (5).

The DC composition differences that we observed with BEN+TBI mirror that of administration of exogenous Flt3L, which robustly expands all DC populations but preferentially increases cDC1s (24, 28). While we did not find elevated plasma levels of Flt3L with BEN+TBI conditioning, we did find a greater expression of the Flt3 receptor, CD135, among DCs conditioned with BEN compared to CY. The Flt3 signaling pathway is intimately involved in the homeostasis, commitment and differentiation of steady state DCs (40, 66, 67). Notably, numerous pre-clinical studies have found that administration of Flt3L *prior* to transplant can alleviate GvHD and enhance GvL, while administration of Flt3L *after* transplant significantly exacerbates GvHD by stimulating donor stem cells to proliferate (24, 28, 68, 69). Thus, BEN+TBI conditioning may prove advantageous over CY+TBI in that it results in enhanced Flt3 signaling, specifically among host cells. Further, the effect of pre-transplant Flt3L has been largely attributed to increased numbers of host CD8α+ cDC1s capable of eliminating antigen-specific donor T-cells (24, 70–72). However, Flt3L also greatly expands pre-cDC1s to the point that nearly half of CD8α- DCs are pre-cDC1s (55). Given the fact that this pre-cDC1 population was only recently characterized, it is possible that the effects of Flt3L on GvHD have been inequitably attributed to CD8α+ cDC1s alone, when perhaps pre-cDC1s also play a significant role. Further investigation is required to fully understand the potential role of pre-cDC1s in alloreactivity and GvHD.

In summary, BEN+TBI conditioning results in a greater number and proportion of murine host pre-cDC1s in a Batf3-independent manner, which is associated with reduced GvHD. We demonstrate that BEN treatment results in host DCs with greater expression of Flt3 receptor, potentially contributing to the skewing of host DCs toward cDC1s. BEN may prove to have significant advantages as a pre-transplant conditioning agent over CY to reduce GvHD and potentially limit viral reactivation.

## Data Availability Statement

The datasets generated for this study are available on request to the corresponding author.

## Ethics Statement

The animal study was reviewed and approved by University of Arizona Institutional Animal Care and Use Committee.

## Author's Note

MM is a Ph.D. Candidate at the University of Arizona. This work is submitted in partial fulfillment of the requirements for the Ph.D.

## Author Contributions

MM designed and performed experiments, analyzed and reviewed data, and wrote the manuscript. JS and EH helped design and perform experiments, reviewed data, and revised the manuscript. JE performed experiments and revised the manuscript. YZ and RS contributed to the experimental design, data interpretation and discussion, and revised the manuscript. EK designed the project, supervised and advised on the implementation and conduction of experiments, reviewed and interpreted data, and co-wrote the manuscript. All authors contributed to the article and approved the submitted version.

## Conflict of Interest

The authors declare that the research was conducted in the absence of any commercial or financial relationships that could be construed as a potential conflict of interest.
