# Supplementary material for: Bendamustine Conditioning Skews Murine Host DCs Toward Pre-cDC1s and Reduces GvHD Independently of Batf3
Source: Front Immunol. 2020 Jul 16;11:1410. doi: 10.3389/fimmu.2020.01410 (PMC7378358; doi:10.3389/fimmu.2020.01410)
Supplement: Supplementary file 1 [file Data_Sheet_1.DOCX]

Supplementary Material

**Supplementary Figure 1**. BALB/c recipient mice were given 40 mg/kg BEN i.v. or 200 mg/kg CY i.p. on day -2 and 400 cGy TBI on day -1. On day 0, mice were transplanted with 10^7^ T-cell depleted bone marrow cells and 3x10^6^ purified total T-cells from C57BL/6 donors. Donor engraftment was determined using flow cytometry by % H2K^b^+ cells in the blood on days 7, 14, 21, 35 and 70 post-transplant. Pooled data from 3 experiments, n=15 mice/group.


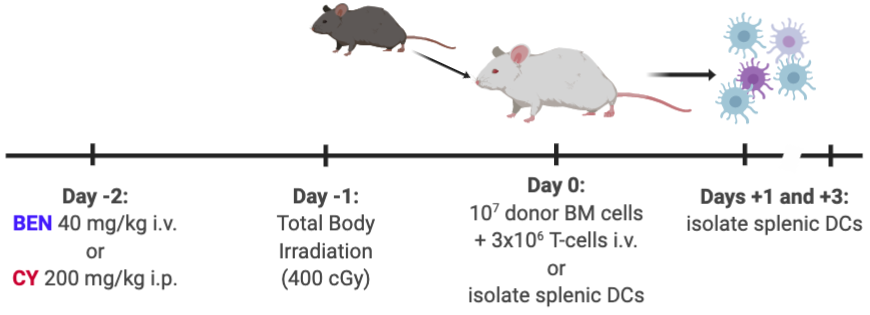


**Supplementary Figure 2.** BALB/c recipient mice were given 40 mg/kg BEN i.v. or 200 mg/kg CY i.p. on day -2 and 400 cGy TBI on day -1. On day 0, either splenic DCs were isolated or mice were transplanted with 10^7^ bone marrow cells and 3x10^6^ purified total T-cells from C57BL/6 donors, then splenic DCs were isolated on day +1 or day +3.

**Supplementary Figure 3.** BALB/c recipient mice were given 40 mg/kg BEN i.v. or 200 mg/kg CY i.p. on day -2 and 400 cGy TBI on day -1. On day 0, spleens were collected, total splenocytes were counted and DCs were isolated and counted. Pooled data from 2 experiments, n=4-6 mice/group.

**Supplementary Figure 4.** BALB/c mice were given 40 mg/kg BEN i.v. or 200 mg/kg CY i.p. on day -2 and 400 cGy TBI on day -1. On day 0, mice were transplanted with 10^7^ bone marrow cells and 3x10^6^ purified total T-cells from C57BL/6 donors. On days +1 and +3, splenic DCs were isolated and analyzed by flow cytometry. Host cells are identified as H2K^b-^. Data from 1 experiment, n=5 mice/group/time point.

| **DC Subset** | **Gating Strategy** |
| --- | --- |
| pDC | CD11c+B220^+^ |
| cDC | CD11c+B220- |
| cDC1 | CD11c+B220-CD8α+ |
| cDC2 | CD11c+B220-SIRPα+CD8α- |
| Pre-cDC1 | CD11c+B220-CD24^high^CD8α- |
| Pre-cDC2 | CD11c+B220-CD24^low^SIRPα+ |
| CD103^+^ cDC1 | CD11c+B220-CD103+ |

**Supplementary Figure 5.** Identifying markers used to characterize the various murine DC subset populations.

**Supplementary Figure 6.** Gating strategies for defined murine DC subsets using isolated splenic pan-DCs.

**Supplementary Figure 7A.** Flow cytometric analysis confirming the isolation purity of the CD8a+ DC Isolation Kit.

**Supplementary Figure 7B.** BALB/c mice received 40 mg/kg BEN or 200 mg/kg CY on day -2 and 400 cGy on day -1. On day 0, splenic CD8α+ cDC1s were isolated by FACS, pooled, and plated in a suppression assay with CellTrace Violet-stained T-cells immediately following stimulation with CD3/CD28 beads at a suppressor to T-cell ratio of 1:1 (middle row) and 1:2 (bottom row). CellTrace Violet dilutions were measure by flow cytometry and proliferation index (PI, boxed) was quantified using ModFit Software. Data is representative of 2 experiments, n=3 mice/group.

**Supplementary Figure 8A.** Flow cytometric analysis confirming the lack of Batf3-dependent CD8α+ cDC1s in Batf3 KO mouse spleens.

**Supplementary Figure 8B.** Flow cytometric analysis confirming the lack of Batf3-dependent CD103+ cDC1s in Batf3 KO mouse spleens.

**Supplementary Figure 8C.** Wild-type BALB/c or transgenic Batf3 KO recipient mice were given 40 mg/kg BEN i.v. or 200 mg/kg CY i.p. on day -2 and 400 cGy TBI on day -1. On day 0, mice were transplanted with 10^7^ bone marrow cells and 3x10^6^ purified total T-cells from C57BL/6 donors. Donor engraftment was determined using flow cytometry by percent H2K^b^+ cells in the blood on days 7, 14, 21, 35 and 70 post-transplant. Data pooled from 3 experiments, n=15-20 mice/group.
